# Three-Dimensional Volumetric Assessment of Diastolic Function by Cardiac Magnetic Resonance Imaging: The Multi-Ethnic Study of Atherosclerosis (MESA)

**DOI:** 10.5935/abc.20170063

**Published:** 2017-06

**Authors:** Marcelo S Nacif, Andre L. C. Almeida, Alistair A Young, Brett R Cowan, Anderson C Armstrong, Eunice Yang, Christopher T Sibley, W. Gregory Hundley, Songtao Liu, Joao AC Lima, David A Bluemke

**Affiliations:** 1 Radiology and Imaging Sciences - National Institutes of Health Clinical Center, Bethesda, MD, USA; 2 Division of Cardiology, Johns Hopkins University School of Medicine, Baltimore, MD, USA; 3 Radiology Department, Universidade Federal Fluminense, Niterói, RJ, Brazil; 4 Auckland MRI Research Group, University of Auckland, Auckland, New Zealand; 5 Department of Internal Medicine and Radiology, Wake Forest University School of Medicine, Winston-Salem, North Carolina, USA; 6 Molecular Biomedical Imaging Laboratory, National Institute of Biomedical Imaging and Bioengineering, Bethesda, MD, USA

**Keywords:** Ventricular Function, Evaluation, Magnetic Resonance, Imaging Three Dimensional, Echocardiography, Three -Dimensional

## Abstract

**Background:**

Cardiac Magnetic Resonance is in need of a simple and robust method for diastolic function assessment that can be done with routine protocol sequences.

**Objective:**

To develop and validate a three-dimensional (3D) model-based volumetric assessment of diastolic function using cardiac magnetic resonance (CMR) imaging and compare the results obtained with the model with those obtained by echocardiography.

**Methods:**

The study participants provided written informed consent and were included if having undergone both echocardiography and cine steady-state free precession (SSFP) CMR on the same day. Guide points at the septal and lateral mitral annulus were used to define the early longitudinal relaxation rate (E'), while a time-volume curve from the 3D model was used to assess diastolic filling parameters. We determined the correlation between 3D CMR and echocardiography and the accuracy of CMR in classifying the diastolic function grade.

**Results:**

The study included 102 subjects. The E/A ratio by CMR was positively associated with the E/A ratio by echocardiography (r = 0.71, p < 0.0001). The early diastolic relaxation velocity by tissue Doppler and longitudinal relaxation rate for the lateral mitral annulus displacement were positively associated (p = 0.007), as were the ratio between Doppler E/e' and CMR E/E' (p = 0.01). CMR-determined normalized peak E (NE) and deceleration time (DT) were able to predict diastolic dysfunction (areas under the curve [AUCs] = 0.70 and 0.72, respectively). In addition, the lateral E/E' ratio showed good utility in identifying diastolic dysfunction (AUC = 0.80). Overall, echocardiography and CMR interobserver and intraobserver agreements were excellent (intraclass correlation coefficient range 0.72 - 0.97).

**Conclusion:**

3D modeling of standard cine CMR images was able to identify study subjects with reduced diastolic function and showed good reproducibility, suggesting a potential for a routine diastolic function assessment by CMR.

## Introduction

the prevalence and cost of treatment of heart failure (HF) in the United States are high. In 2008, this condition was estimated to affected 5.3 million adults and was associated with a total spending of 34.8 billion dollars.^1,2^ Approximately 50% of the patients were reported to have diastolic HF.^1,2^ Diastolic dysfunction is an increasingly recognized component of a variety of diseases of the myocardium,^3,4^ and its recognition is necessary for patient management.^5^

Echocardiography is currently used as the standard of reference to evaluate diastolic dysfunction.^6-10^ With cardiac magnetic resonance (CMR) imaging, diastolic function is assessed using special pulse sequences such as phase-contrast analysis or myocardial tissue tagging.^5,6,8,11-16^ These assessments require additional time and software for acquisition and analysis. As a result, the diastolic assessment with CMR is not routinely applied.^5,17,18^ Thus, CMR is in need of a simple and robust method for diastolic function assessment that can be done with routine protocol sequences.

A three-dimensional (3D) model of myocardial function has been developed to assess the myocardial function based on standard steady-state free precession (SSFP) CMR cine images.^19^ A model-based analysis of the systolic function is relatively fast (~15 minutes per CMR study) and allows extraction of time-varying function parameters that may characterize the diastolic function.^19-23^

Thus, the purpose of this study was to perform an intraindividual analysis to develop and validate a 3D model-based volumetric assessment of diastolic function using CMR imaging and compare the results obtained with this model with those obtained by echocardiography.

## Methods

### Study population

**Figure 1:**
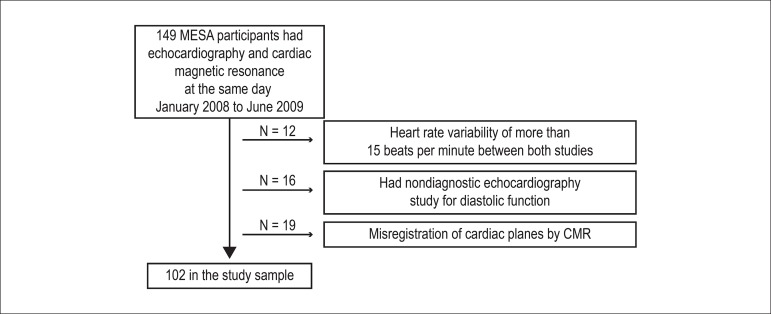
Flowchart of the study population. Abbreviation: CMR: cardiac magnetic resonance.

The study included participants who underwent both echocardiography and CMR between 2008 and 2009 in a substudy of the Multi-Ethnic Study of Atherosclerosis (MESA) at the Johns Hopkins Hospital. Details of the MESA study have been previously described.^24^ In brief, 1096 participants free of clinically apparent cardiovascular disease and aged 45-84 years, were enrolled at the Baltimore field center at baseline in 2000-2002. A total of 149 consecutive participants were invited to participate in the CMR-echocardiography substudy. Participants were excluded if they had not undergone both studies at the same day; if they had a heart rate variability of more than 15 beats per minute between both studies, severe mitral annular calcification or mitral valve regurgitation; or if the qualitative assessments of the left ventricular (LV) function was impaired by arrhythmias or poor image quality by either modality (Figure 1). The study was approved by the local ethics committee, and all subjects gave a written informed consent for participation.

Since this study included a correlation between echocardiography and CMR, not all variables were used in the analysis. We will describe the variables that can be acquired by echocardiography and the 3D model-based volumetric assessment of diastolic function using CMR.

### Echocardiography

Echocardiograms were obtained by expert sonographers according to the recommendations of the American Society of Echocardiography (ASE).^10^ The examinations were reviewed offline by two readers. Readers 1 (A.L.C.A.) and 2 (A.C.A.) had 20 and 5 years of experience, respectively, in reading echocardiograms. Two-dimensional (2D) echocardiograms were recorded using an Aplio scanner (Toshiba Medical Systems Corp, Tochigi, Japan). The images were acquired from an LV apical four-chamber view. Image acquisitions were performed using B-mode harmonic images adjusting transducer frequencies (1.7-3.5 MHz), frame rate (40-80 frames per second), focus, sector width (as narrow as possible), sector depth (minimal), and gain, in order to optimize myocardial image quality. The images were digitally recorded, stored on compact discs, and transferred to a computer terminal for post processing.

**Mitral inflow velocities:** All Doppler measurements were assessed according to the ASE recommendations.^25^ From the transmitral recordings, the following measurements were carried out:

- transmitral early peak filling velocity during diastole (early peak filling rate [E]), in centimeters per second;
- (transmitral late peak atrial filling velocity during diastole [peak atrial velocity [A]), in centimeters per second;
- time elapsed between E and the point where the extrapolation of the deceleration slope of the E velocity crosses the zero baseline (deceleration time [DT]), in milliseconds;
- time elapsed between the systolic peak to E (time to peak E [relative TPE]), in milliseconds;
- time elapsed between the systolic peak to A (time to peak A [relative TPA], in milliseconds.

**Tissue Doppler measurement of mitral annular velocity:** Pulsed wave tissue Doppler imaging (TDI) was performed in the apical views to acquire the mitral annular velocities according to the ASE recommendations.^25^ The sample volume was placed in the ventricular myocardium immediately adjacent to the mitral annulus in the septal and lateral walls. With this method, the early diastolic myocardial relaxation velocity (e'; cm/s), as the annulus ascends away from the apex, was assessed in this study.

### Cardiac magnetic resonance

Cine-CMR images were acquired on a 1.5 T scanner (Avanto, Siemens, Malvern, PA, USA) using a 2D SSFP acquisition in vertical long-axis, horizontal long-axis, and short-axis orientations with the following parameters: TE 1.16 ms, TR 3.2 ms, flip angle 60°, receiver bandwidth ±1220 kHz, FOV 36 cm, slice thickness 8 mm, slice gap 2 mm, acquisition matrix 205 × 256, number of averages = 1, number of frames = 30. The mean reconstructed temporal resolution (R-R interval/number of cardiac phases) was 30.43 ± 5.44 ms.

CMR images were analyzed using a research version of the CIM 6.2 program modified to assess diastolic function (Auckland MRI Research Group, University of Auckland, New Zealand).^19^ CMR image analyses were done by two readers accredited by the Auckland MRI Research Group. Readers 1 (M.S.N.) and 2 (E.Y.) had 7 years and 1 year of experience, respectively, in reading CMR.

**Figure 2:**
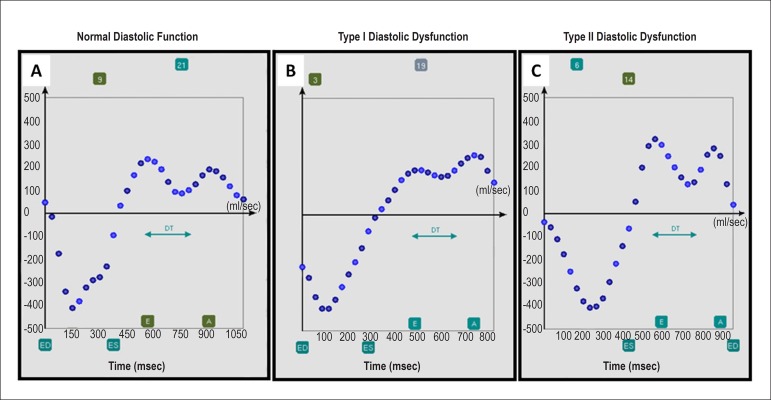
Screenshots of different diastolic function examples using the program CIM. A) Normal, B) impaired, and C) reduced. The following measurements were assessed: end-systole (ES), end-diastole (ED), early peak filling rate (E), atrial peak filling rate (A), and deceleration time (DT). All timing measurements were defined semiautomatically with manual correction with the observer using a slider on the time/rate curve.

**Time-volume curve:** All timing measurements were defined semiautomatically with manual correction with the observer using a slider on the time/rate curve (Figure 2). The following measurements were assessed:

- diastolic volume recovery (DVR), defined as the time from end-systole (ES) to the time at which the volume has filled to 80% of the stroke volume (msec);
- E (mL/sec), the first maximum filling rate detected after ES. Peak E was also divided by the end-diastolic volume (EDV) to generate a normalized peak E filling rate (NE). Additional measurements included:
- relative time to early peak filling rate (RTPE) (msec), the trigger time to peak E from the ES phase;
- A (mL/sec), the second peak filling rate after ES. Peak A was also indexed by EDV to generate a normalized peak A filling rate (NA);
- relative time of atrial peak filling rate (RTPA; msec), the trigger time to peak A from the ES phase; and
- DT (msec), or the time delay of E subtracted from the E wave downslope intersecting the baseline.

**Figure 3:**
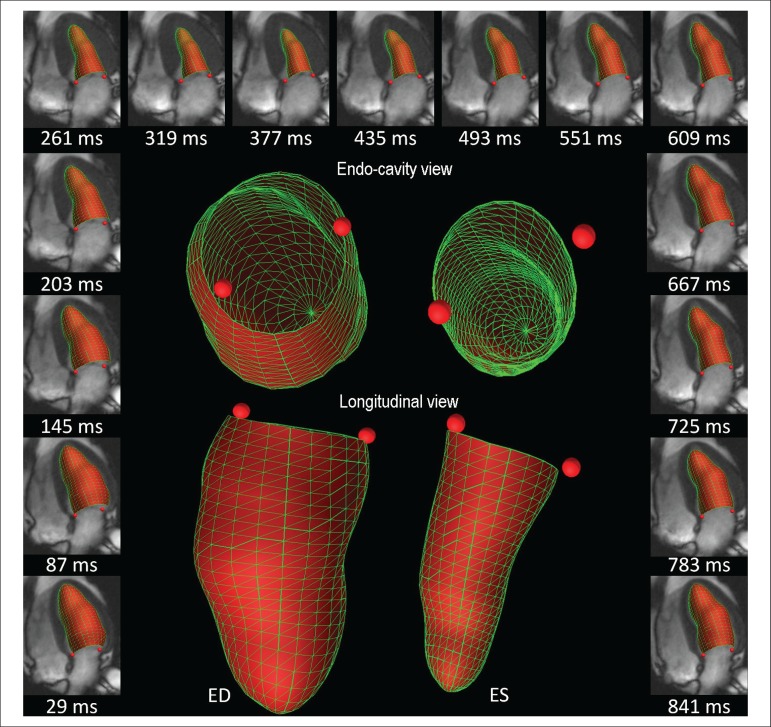
Three-dimensional displays of the model fits throughout the cardiac cycle in one R-R interval of 870 ms (outside images) for volume- and derivative-curve assessment (mL/s). Septal and lateral guide points motion can be evaluated through time, calculating the distance between the defined point and the model apex for myocardial longitudinal relaxation rate (mm/s). The endocardial surface is shaded in red and drawn with green lines.

Guide points at the junction of the LV wall with the septal mitral annulus and at the junction of the LV wall with the lateral mitral annulus in the four-chamber view were used to define g) E' septal, and h) E' lateral, respectively. The ratio between E and E' was also calculated (Figure 3).

Note that CMR rates are expressed as volume (mL) per unit of time, whereas echocardiographic parameters are expressed as distance (cm) per unit of time. However, CMR-derived E' is expressed as a linear velocity similar to its echocardiographic correlate.

### Data and statistical analysis

The diastolic function classification used three echocardiographic parameters recommended by the ASE for this purpose:

1. septal e' < 8 cm/s,
2. lateral e'< 10 cm/s, and
3. ratio between average E and average e' ≥ 10.^25^ If all three criteria were present, the diastolic function was rated as type II (reduced). If only two criteria were present or one criteria plus LV hypertrophy, the diastolic function was rated as type I (impaired). The LV mass was assessed by echocardiography and divided by the body surface area to define the LV mass index (LVMi). LV hypertrophy was defined as an LVMi > 115 g/m^2^ for men and > 95 g/m^2^ for women, as recommended by the ASE.^26^

Data are presented as mean ± standard deviation (SD) for continuous variables and as percentage for categorical variables. Multiple comparisons were tested by one-way analysis of variance (ANOVA) with *post hoc* Bonferroni correction. Fisher's exact test was used to examine the differences between proportions. As the variables were normally distributed, linear regression analysis was performed using Pearson's correlation coefficient (r) and setting echocardiography as the predictor variable and CMR as the dependent variable. We used Bland-Altman to compare variables with the same units. However, in many cases, the CMR surrogates for echocardiographic parameters were represented in different units, so the Bland-Altman analysis was inappropriate.

Receiver operating characteristic (ROC) curve analysis was used to identify the diagnostic performance of CMR in predicting diastolic dysfunction. This was achieved by using the group with reduced diastolic function assessed by echocardiography as the "true positive" surrogate marker for diastolic dysfunction in this population, compared with the group with normal function as the "true negative" (area under the curve [AUC] ≥ 0.5 to < 0.7 = poor fit, AUC ≥ 0.7 to < 0.9 = good fit, and AUC ≥ 0.9 to 1.0 = excellent fit).

Intraobserver and interobserver agreements were assessed using intraclass correlation coefficient (ICC) with a two-way random model (ICC < 0.40 = poor agreement, ICC ≥ 0.40 to 0.75 = fair to good agreement, ICC > 0.75 = excellent agreement).

The statistical analysis was performed using Stata, version 12.0 (StataCorp LP, College Station, Texas, USA). A p value < 0.05 was considered significant.

## Results

A total of 102 participants met the inclusion criteria (Figure 1). On echocardiography, the diastolic function was classified as normal in 66 (64.7%) patients, impaired in 21 (20.6%), and reduced in 15 (14.7%) of them. The mean duration of the CMR analysis (systolic and diastolic function) was 18.3 ± 4.5 minutes. Note that the CMR analysis also yields parameters such as LV volume and mass, since the analysis is performed over the full cardiac cycle. The mean duration of the echocardiographic analysis (diastolic function only) was 4.6 ± 0.6 minutes (p < 0.0001 compared with the CMR analysis). Reduced diastolic function was more frequent in diabetic and hypertensive participants. Major variables, *e.g.,* age, gender, body mass index (BMI), systolic blood pressure (SBP), LV mass, EDV, and heart rate, showed no significant variance between groups. The characteristics of the subjects and the clinical data related to their LV function are summarized in Table 1.

**Table 1:** Population characteristics by diastolic function grades

	Normal n = 66 (64.70%)	Type I n = 21 (20.60%)	Type II n = 15 (14.70%)	p value
Age (years)	66.8 ± 8.9	65.5 ± 7.5	64.4 ± 9.7	0.60
45 to 64 years	24 (36.3)	11 (52.3)	7 (46.6)	0.48*
65 to 84 years	42 (63.6)	10 (47.6)	8 (53.3)	0.48*
Gender (male)	26 (39.3)	7 (33.3)	6 (40.0)	0.91
**Race**				
White, Caucasian	41 (62.0)	11 (55.0)	6 (40.0)	0.25*
Black, African-American	25 (38.0)	10 (45.0)	9 (60.0)	0.25*
Weight (kg)	77.5 ± 15.1	80.3 ± 19.4	80.3 ± 22.2	0.73
Height (cm)	168.0 ± 9.4	166.0 ± 11.2	166.1 ± 9.7	0.65
BMI (kg/m^2^)	28.0 ± 4.4	29.1 ± 5.7	28.2 ± 7.1	0.71
BSA	1.8 ± 0.2	1.8 ± 0.2	1.8 ± 0.2	0.89
**Smoking status**				
Never	27 (40.9)	9 (42.8)	5 (33.3)	0.37*
Former	33 (50.0)	12 (57.1)	7 (46.6)	0.37*
Current	6 (9.0)	0 (0.0)	3 (20.0)	0.37*
Systolic blood pressure (mmHg)	121.8 ± 18.7	119.8 ± 14.6	121.3 ± 25.9	0.91
Diastolic blood pressure (mmHg)	71.2 ± 11.2	66.3 ± 10.6	69.1 ± 10.7	0.21
Hypertension (%)	33 (50.0)	7 (33.3)	9 (60.0)	0.28*
Any hypertension medication	31 (46.9)	6 (28.5)	9 (60.0)	0.20*
Diabetes (%)	3 (4.5)	2 (9.5)	3 (20.0)	0.11*
Triglycerides (mg/dL)	111.9 ± 60.5	100.3 ± 67.3	101.3 ± 54.8	0.68
LDL cholesterol (mg/dL)	111.5 ± 32.3	109.1 ± 34.2	112.4 ± 42.9	0.95
HDL cholesterol (mg/dL)	58.9 ± 18.4	62.4 ± 24.9	52.6 ± 12.0	0.33
Total cholesterol (mg/dL)	192.8 ± 38.5	191.5 ± 38.8	185.2 ± 52.7	0.81
Metabolic syndrome	21 (31.8)	4 (19.0)	2 (13.0)	0.26*
**Echocardiographic measurements**				
Heart rate (beats/min)	64.8 ± 9.6	65.0 ± 9.4	62.6 ± 5.7	0.66
End-diastolic diameter (mm)	4.4 ± 0.5	4.5 ± 0.5	4.6 ± 0.4	0.44
Diastolic septal thickness (mm)	1.0 ± 0.2	1.0 ± 0.1	0.9 ± 0.1	0.18
Diastolic inferolateral thickness (mm)	0.9 ± 0.1	0.9 ± 0.1	0.9 ± 0.1	0.66
**CMR measurements**				
Heart rate (beats/min)	65.2 ± 10.4	66.4 ± 9.7	61.6 ± 5.5	0.31
Ejection fraction (%)	69.0 ± 7.3	70.7 ± 7.0	70.6 ± 10.2	0.51
End-diastolic volume (mL)	106.8 ± 24.4	110.6 ± 28.7	99.6 ± 22.1	0.43
End-systolic volume (mL)	33.8 ± 13.6	33.5 ± 14.2	28.8 ± 10.6	0.42
LV mass (g)	124.8 ± 34.4	132.5 ± 38.2	121.8 ± 26.2	0.59
Stroke volume (mL)	73.0 ± 15.1	76.5 ± 18.0	73.1 ± 17.5	0.67

BMI: body mass index; BSA: body surface area; CMR: cardiac magnetic resonance; LV: left ventricular. Note:

* Fisher’s exact test was used to compare proportions between diastolic severity grades.

Echocardiographic parameters showed increasing mean values in association with diastolic dysfunction severity (p < 0.05, Table 2). However, A alone showed no statistically significant difference between groups. NE and DT obtained from derivative volume-curves by CMR showed trends toward diastolic dysfunction severity similar to those obtained by echocardiography (p < 0.05). The E/A ratio by CMR was 1.10 ± 0.38 in the normal group, and was lower in the impaired group (1.01 ± 0.26) and higher in the reduced diastolic function group (1.33 ± 0.45, p = 0.03). All other variables showed no difference between groups (Table 2).

**Table 2:** Diastolic measurements by echocardiography and cardiac magnetic resonance

	Normal n = 66 (64.70%)	Type I n = 21 (20.60%)	Type II n = 15 (14.70%)	p value
**Echocardiography**				
**Mitral inflow velocities**				
E (cm/s)	74.53 ± 16.43	74.89 ± 20.76	87.68 ± 20.94	0.03
DT (ms)	220.14 ± 45.19	247.95 ± 76.70	258.4 ± 77.69	0.03
A (cm/s)	77.66 ± 19.59	75.40 ± 19.55	77.18 ± 23.09	0.90
E/A	0.99 ± 0.24	1.02 ± 0.22	1.23 ± 0.47	0.01
**Tissue Doppler velocities**				
**Septal**				
e’ (cm/s)	9.38 ± 1.69	8.33 ± 2.12	6.00 ± 1.26	<0.0001
E/e’	8.20 ± 2.24	9.23 ± 2.22	15.37 ± 5.91	<0.0001
**Lateral**				
e’(cm/s)	11.61 ± 2.45	8.23 ± 1.68	6.97 ± 1.80	<0.0001
E/e’	6.65 ± 1.82	9.37 ± 3.22	13.36 ± 5.21	<0.0001
**Mean**				
e’ (cm/s)	10.47 ± 1.59	8.27 ± 1.45	6.48 ± 1.44	<0.0001
E/e’	7.25 ± 1.78	9.12 ± 2.06	14.19 ± 5.40	<0.0001
**CMR**				
**Volume-curves**				
E (mL/s)	189.30 ± 66.39	206.30 ± 62.58	213.60 ± 71.67	0.33
NE (s^-1^)	1.77 ± 0.46	1.89 ± 0.50	2.11 ± 0.43	0.03
DT (ms)	186.61 ± 43.94	211.08 ± 43.75	218.37 ± 42.59	0.01
TPE (ms)	504.86 ± 82.41	493.46 ± 68.75	517.54 ± 37.80	0.63
A (mL/s)	181.13 ± 72.08	211.09 ± 75.17	164.73 ± 43.96	0.11
NA(s^-1^)	1.70 ± 0.53	1.98 ± 0.76	1.71 ± 0.58	0.16
TPA (ms)	837.27 ± 193.40	861.57 ± 155.17	866.00 ± 115.64	0.78
E/A	1.10 ± 0.38	1.01 ± 0.26	1.33 ± 0.45	0.03
DVR (ms)	535.32 ± 117.96	542.44 ± 122.45	516.08 ± 78.16	0.80
**Longitudinal relaxation rate**				
**Septal**				
E’ (mm/s)	75.35 ± 24.49	66.49 ± 25.31	58.22 ± 24.11	0.03
E/E’ (mL/mm)	2.64 ± 0.96	3.45 ± 1.60	4.65 ± 3.38	0.0002
**Lateral**				
E’ (mm/s)	82.36 ± 26.14	70.88 ± 28.45	61.06 ± 27.73	0.01
E/E’ (mL/mm)	2.40 ± 0.83	3.32 ± 1.80	4.52 ± 3.54	0.0001
**Mean**				
E’ (mm/s)	78.86 ± 24.85	68.69 ± 26.26	59.64 ± 25.45	0.02
E/E’ (mL/mm)	2.50 ± 0.87	3.33 ± 1.53	4.55 ± 3.44	0.0001

E: early peak filling rate; DT: deceleration time; A: atrial peak filling rate; E/A:E/A ratio; e’: early diastolic myocardial relaxation velocity; E/e’: E/e’ ratio; NE: normalized peak E filling rate; NA: normalized peak A filling rate; DVR: diastolic volume recovery; E’: early longitudinal relaxation rate; CMR: cardiac magnetic resonance, TPE: time to peak E; TPA: time to peak A.

Tissue Doppler velocities by echocardiography assessed e' and the E/e' ratio. In all regions (septal and lateral mitral annulus), e' showed significantly decreased mean values in the normal diastolic function group (e' lateral = 11.6 ± 2.4 cm/s) and in the reduced diastolic function group (e' lateral = 6.9 ± 1.8 cm/s, p < 0.05). Also, E/e' increased from the group with a normal diastolic function to the one with reduced diastolic function (6.65 ± 1.8 and 13.3 ± 5.2, respectively, p < 0.0001). Compared with CMR, E' and E/E' showed similar trends toward worse diastolic function for both septal and lateral walls (p < 0.05 and p < 0.001, respectively) (Table 2).

**Figure 4:**
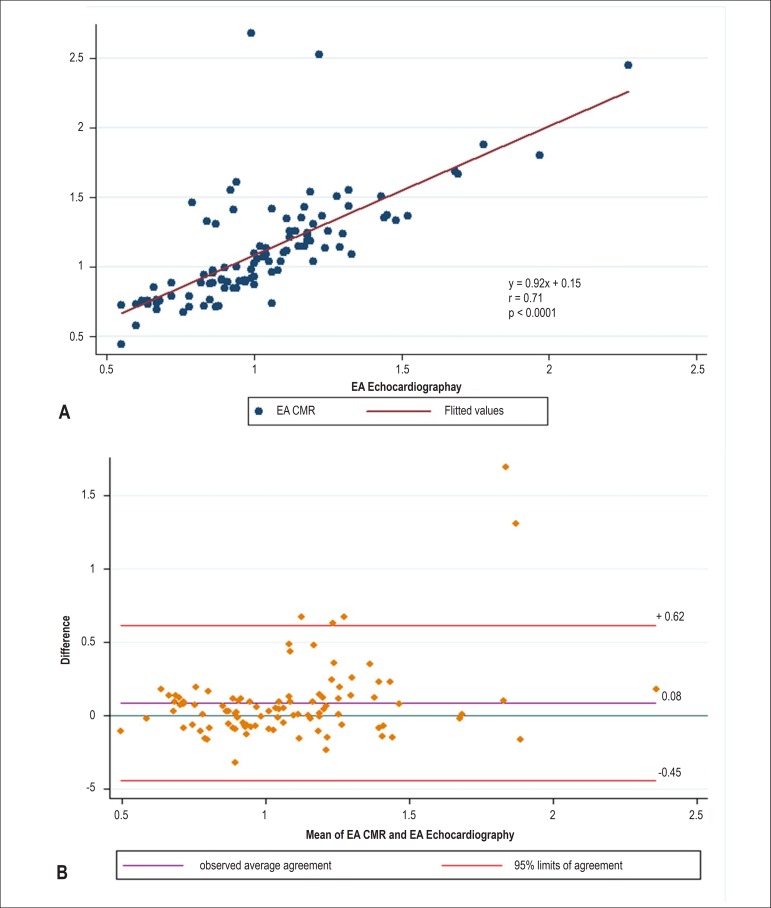
Results obtained using cardiac magnetic resonance (CMR) three-dimensional volume-curve and echocardiography Doppler mitral valve inflow. The ratio between the early peak filling (E) and atrial peak filling rate (A) using velocity (cm/s) by echocardiography and flow (mL/s) by CMR. (A) Linear regression and Pearson's correlation; (B) Bland-Altman analysis.

Table 3 highlights the associations between the diastolic function measured by echocardiography and CMR. E/A ratios on echocardiography were positively associated with E/A ratios on CMR (r = 0.71, p < 0.0001). The 95% limits of agreement between the two methods were -0.45% to +0.62%. A small bias (0.081%) toward a higher E/A ratio by CMR was detected (Figure 4).

**Table 3:** Associations between measures of diastolic function by echocardiography and cardiac magnetic resonance (n = 102)

Echocardiography	CMR	Pearson’s correlation coefficient (r)	p value
**Mitral inflow velocities**	**Volume-curves**		
E (cm/s)	E (mL/s)	0.06	0.51
E (cm/s)	NE (s^-1^)	0.1	0.18
A (cm/s)	A (mL/s)	0.22	0.01
A (cm/s)	NA (s^-1^)	0.28	0.003
E/A	E/A	0.71	< 0.0001
**Tissue Doppler**	**Longitudinal relaxation rate**		
**Septal**	**Septal**		
e’ (cm/s)	E’ (mm/s)	0.11	0.26
E/e’	E/E’ (mL/mm)	0.11	0.30
**Lateral**	**Lateral**		
e’ (cm/s)	E’ (mm/s)	0.26	0.007
E/e’	E/E’ (mL/mm)	0.24	0.01
**Mean**	**Mean**		
e’ (cm/s)	E’ (mm/s)	0.22	0.02
E/e’	E/E’ (mL/mm)	0.17	0.07

CMR: cardiac magnetic resonance; early peak filling rate; A: atrial peak filling rate; e’: early diastolic myocardial relaxation velocity; NE: normalized peak E filling rate; NA: normalized peak A filling rate; E/A: E/A ratio; E’: early longitudinal relaxation rate. Echocardiography corresponds to Doppler echocardiography.

Values of e' by tissue Doppler and E' for the lateral mitral annulus displacement were positively correlated (r = 0.26, p = 0.007), as were E/e' by CMR and echocardiography (r = 0.24, p = 0.01). However, both septal measurements were not correlated (p > 0.05).

### Prediction of reduced diastolic function by cardiac magnetic resonance

Table 4 shows the ROC curve analysis for reduced diastolic function for all CMR parameters. CMR-determined NE and DT were able to predict diastolic dysfunction (AUCs = 0.70 and 0.72, respectively). In addition, the lateral E/E' ratio appeared to be useful in the classification of diastolic dysfunction (AUC = 0.80) (Table 4).

**Table 4:** Prediction of reduced diastolic function by cardiac magnetic resonance (n = 81)

CMR	Area under the ROC curve	p value
**Volume-curves**		
E (mL/s)	0.60	0.21
NE (s^-1^)	0.70	0.008
DT (ms)	0.72	0.01
A (mL/s)	0.53	0.37
NA (s^-1^)	0.48	0.92
DVR (ms)	0.51	0.57
E/A	0.66	0.05
**Longitudinal relaxation rate**		
**Septal**		
E’ (mm/s)	0.67	0.01
E/E’ (mL/mm)	0.76	0.0003
**Lateral**		
E’ (mm/s)	0.70	0.0004
E/E’ (mL/mm)	0.80	< 0.0001
**Mean**		
E’ (mm/s)	0.69	0.006
E/E’ (mL/mm)	0.78	0.0001

CMR: cardiac magnetic resonance; ROC: receiver operating characteristic; E: early peak filling rate; NE: normalized peak E filling rate; DT: deceleration time; A: atrial peak filling rate; NA: normalized peak A filling rate; DVR: diastolic volume recovery; E/A: E/A ratio; E’: early longitudinal relaxation rate; E/E’: E/E’ ratio.

### Diastolic time periods and cardiac cycle duration

No significant differences were detected in relative TPE and RTPA values obtained by CMR compared with those obtained by echocardiography (mean RTPA: 183.3 ± 47.32 ms *versus* 181.5 ± 27.45 ms, respectively, p = 0.90; mean TPE: 544.32 ± 145.62 ms *versus* 550.77 ± 196.19 ms, respectively, p = 0.91). The cardiac cycle duration (R-R interval) was also not significantly different by CMR *versus* echocardiography (mean 943.65 ± 135.11 ms *versus* 944.77 ± 135.42 ms, respectively, p = 0.95).

### Interobserver and intraobserver agreements

Overall, echocardiography and CMR interobserver and intraobserver agreements were excellent (Table 5). The mean ICC for measurements by echocardiography was excellent (0.89) and slightly higher than those obtained by CMR (0.86).

**Table 5:** Intraobserver and interobserver agreement (n = 20)

	Intraclass correlation coefficient (ICC)	Bias	95% limits of agreement
**Echocardiography R1 *versus* R2**			
Mitral inflow velocities			
E (cm/s)	0.93	-1.66	-11.70 to 8.36
DT (ms)	0.84	9.84	-38.67 to 58.36
A (cm/s)	0.95	-1.12	-14.84 to 12.59
**Tissue Doppler velocities**			
**Septal**			
e’ (cm/s)	0.85	0.42	-1.84 to 2.68
**Lateral**			
e’ (cm/s)	0.89	-0.37	-1.76 to 2.49
**Echocardiography R1 *versus *R1**			
**Mitral inflow velocities**			
E (cm/s)	0.95	-1.39	-9.22 to 6.44
DT (ms)	0.72	6.77	-62.40 to 75.96
A (cm/s)	0.96	-0.22	-12.85 to 12.39
**Tissue Doppler velocities**			
**Septal**			
e’ (cm/s)	0.89	0.28	-1.78 to 2.35
**Lateral**			
e’ (cm/s)	0.92	-0.59	-2.06 to 0.86
**CMR R1 *versus* R2**			
**Volume-curves**			
E (mL/s)	0.84	2.54	-79.77 to 84.86
DT (ms)	0.77	-21.52	-81.75 to 38.70
A (mL/s)	0.82	22.89	-51.20 to 97.00
**Longitudinal relaxation rate**			
**Septal**			
E’ (mm/s)	0.75	-4.90	-32.59 to 22.63
**Lateral**			
E’ (mm/s)	0.89	-5.48	-25.24 to 14.27
**CMR R1 *versus* R1**			
**Volume-curves**			
E (mL/s)	0.97	-1.36	-33.73 to 31.00
DT (ms)	0.84	12.93	-28.46 to 28.46
A (mL/s)	0.96	-15.51	-64.82 to 33.79
**Longitudinal relaxation rate**			
**Septal**			
E’ (mm/s)	0.85	-2.93	-23.91 to 18.03
**Lateral**			
E’ (mm/s)	0.94	-4.11	-18.93 to 10.70

Note: R1: reader 1 and R2: reader 2. E: early peak filling rate; DT: deceleration time; A: atrial peak filling rate; e’: early diastolic myocardial relaxation velocity; E’: early longitudinal relaxation rate.

## Discussion

The purpose of this study was to evaluate the role of cine CMR for diastolic function assessment and compare values obtained with this method with those obtained with echocardiography. Using a relatively fast and reproducible method, CMR-derived parameters were shown to be comparable to those obtained by echocardiography, with good correlations. Importantly, this study demonstrated that CMR was capable of identifying diastolic dysfunction in most patients with diastolic dysfunction detected by echocardiography. This suggests a role for CMR in the assessment of LV diastolic function in the general population.

Echocardiography has long been used to evaluate diastolic dysfunction. The combination of mitral inflow velocity curves and tissue Doppler velocities of the mitral annulus are known to provide better estimates of LV filling pressures than other methods.^27^ Although routinely reported by echocardiography, diastolic function by CMR is usually not routinely assessed due to the requirement of additional phase contrast or tagged sequences, as well as separate post processing. Automated segmentation of LV volumes for all temporal phases holds the potential to rapidly assess diastolic filling patterns;^28^ however, this method alone only provides partial information regarding the diastolic physiology needed to differentiate all degrees of diastolic dysfunction severity.

Recently, CMR software innovations^19,29,30^ have allowed the assessment of similar parameters using SSFP cine CMR with 3D post processing. HF with preserved ejection fraction is increasing in incidence and has a high clinical relevance,^8^ although a clear consensus for its diagnosis has yet to be established.^31^ In this study, we decided to follow the ASE recommendations^25^ to delineate normal *versus* reduced diastolic function groups.

CMR is considered a reference standard for ventricular systolic function, including the analysis of regional wall motion, mass, and volumes, and estimation of ejection fraction.^32^ The assessment of diastolic function by CMR is usually not routinely performed in our clinical practice. CMR diastolic assessment typically requires an increased scan time for image acquisition (*e.g.*, additional phase-contrast sequences), as well as a tedious imaging post-processing analysis. Automated segmentation of LV volumes for all temporal phases holds the potential to assess rapidly diastolic filling patterns;^28^ however, this method relies on a sequential cross-sectional analysis that only provides partial information regarding the diastolic physiology needed to differentiate all degrees of diastolic dysfunction severity.

In our study, we were able to overcome several limitations of CMR diastolic function analysis using a new 3D method with an average analysis time of fewer than 20 minutes with no need to add more sequences in our routine protocol. In our experience, this analysis time is comparable to that obtained for full 3D volumetric assessment of systole alone. For CMR, it appears that E/E', NE, DT, and E/A were the most useful parameters obtained from time-volume curves. When the longitudinal shortening was measured, both septal and lateral measurements were able to categorize diastolic dysfunction. However, E' at the lateral wall was shown to be more reproducible and easily measured by CMR. By comparison, septal E' had lower reader reproducibility.

This study had several limitations. Echocardiography was used as the reference standard, but reader variability and diastolic classification are known to be imperfect with this method.^31^ The correlation between LV mitral valve inflow velocities and time-volume curves from CMR represents different physiological processes. The time-volume curves from CMR should not be adversely affected by mitral valve disease or angle of acquisition.^9,25^ In this study population, hemodynamic data were not available. In addition, our study population did not include subjects with restrictive cardiomyopathy. CMR time-volume curves represent the average of several cardiac cycles, whereas echocardiography shows peak values for each cardiac cycle. Finally, although a good correlation of echocardiographic and CMR data appears to be present, outcome data is still needed to validate further the CMR approach.

## Conclusion

The 3D CMR method was relatively fast, reproducible, and successfully applied to routine SSFP cine CMR data. CMR was able to identify most patients with reduced diastolic function identified by echocardiography. This suggests a role for CMR in the assessment of LV diastolic function in the general population and in patients with mild and moderate diastolic dysfunction.
